# How do adolescents talk about self-harm: a qualitative study of disclosure in an ethnically diverse urban population in England

**DOI:** 10.1186/1471-2458-13-572

**Published:** 2013-06-11

**Authors:** Emily Klineberg, Moira J Kelly, Stephen A Stansfeld, Kamaldeep S Bhui

**Affiliations:** 1 Centre for Psychiatry, Wolfson Institute of Preventive Medicine, Queen Mary University of London, Barts& The London School of Medicine and Dentistry, London, UK; 2 Blizard Institute, Queen Mary University of London, Barts& The London School of Medicine and Dentistry, London, UK; 3 Academic Department of Adolescent Medicine, The Children’s Hospital at Westmead, Locked Bag 4001, Westmead, NSW 2145, Australia

**Keywords:** Self-harm, Help seeking, Adolescent, Qualitative methods, Self-injury, Ethnicity

## Abstract

**Background:**

Self-harm is prevalent in adolescence. It is often a behaviour without verbal expression, seeking relief from a distressed state of mind. As most adolescents who self-harm do not seek help, the nature of adolescent self-harm and reasons for not disclosing it are a public health concern. This study aims to increase understanding about how adolescents in the community speak about self-harm; exploring their attitudes towards and experiences of disclosure and help-seeking.

**Methods:**

This study involved 30 qualitative individual interviews with ethnically diverse adolescents aged 15–16 years (24 females, 6 males), investigating their views on coping with stress, self-harm and help-seeking, within their own social context in multicultural East London. Ten participants had never self-harmed, nine had self-harmed on one occasion and 11 had self-harmed repeatedly. Verbatim accounts were transcribed and subjected to content and thematic analysis using a framework approach.

**Results:**

Self-harm was described as a complex and varied behaviour. Most participants who had self-harmed expressed reluctance to talk about it and many had difficulty understanding self-harm in others. Some participants normalised self-harm and did not wish to accept offers of help, particularly if their self-harm had been secretive and ‘discovered’, leading to their referral to more formal help from others. Disclosure was viewed more positively with hindsight by some participants who had received help. If help was sought, adolescents desired respect, and for their problems, feelings and opinions to be noticed and considered alongside receiving treatment for injuries. Mixed responses to disclosure from peers, family and initial sources of help may influence subsequent behaviour and deter presentation to services.

**Conclusions:**

This study provides insight into the subjective experience of self-harm, disclosure and help-seeking from a young, ethnically diverse community sample. Accounts highlighted the value of examining self-harm in the context of each adolescent’s day-to-day life. These accounts emphasised the need for support from others and increasing awareness about appropriate responses to adolescent self-harm and accessible sources of help for adolescents.

## Background

Self-harm is a frequent, significant and concerning public health issue [1-4]. Community-based research indicates around 7% of 15–16 year olds have self-harmed in the last year [3,5-7]. Self-harm is described as a behavioural expression of psychological distress to seek relief from a “terrible state of mind” [8,9]. Although it has been shown to be a predictor of later suicide [2], and some self-harm has suicidal ideation, there is evidence that adolescent self-harm may be part of a repertoire of experimental adolescent behaviours [10]. It often takes the form of “non-suicidal-self- injury” (NSSI) and is associated with diagnostic heterogeneity [11]. As this is a non-verbal expression of emotion, it follows that people who self-harm may find it challenging to talk about self-harm, and may not disclose it or seek help.

There are low rates of help-seeking among adolescents who self-harm. Community-based studies report that only 10-13% of adolescents who had self-harmed presented to hospital [3,12,13]. Use of more lethal methods in self-harm and intention to die have been associated with receiving help from health services [12], however, other people are often involved in facilitating that help provision. Help-seeking is poorer in adolescents who have greater depressive symptoms [14] and higher suicidal ideation [15], indicating that a lack of help-seeking could function as a risk factor for adverse outcomes. Attitudes to help-seeking in young people who self-harm remain somewhat unclear. A “staged” model of help-seeking [16] proposes that prior to seeking help, adolescents would need to view self-harm as a legitimate problem, believe they might be helped, locate a help source, and be motivated to follow through seeking help [17]. Adolescents are likely to seek informal help initially, telling friends or family rather than approaching health services [7,12,13,17]. Barriers to help-seeking for self-injury or depressive symptoms may include not recognising any concerns, or not acting upon concerns if they were recognised [18]. Other barriers may include self-stigma or perceived stigma from others about the harm or help-seeking [19,20], not knowing where to seek help or not being able to find accessible youth-friendly services [21], or it not being perceived as culturally appropriate to seek help [22,23]. Responses from others following disclosure [16], and negative experiences having sought help [24] may influence subsequent help-seeking.

Investigating personal accounts of self-harm and help-seeking can educate health professionals about the sensitivities and complexities they will encounter when gathering information during clinical assessments and during therapies. Personal accounts can also help to inform the shape of future services [25]. Further understanding about this behaviour which commonly remains below a clinical threshold can inform public health policy about prevention and appropriate responses to adolescent self-harm at a community level. This issue is highlighted by the inclusion of hospital admissions for self-harm as an indicator in the Public Health Outcomes framework for England, 2013–2016 [26] and reference to self-harm within the guidance strategy on Preventing Suicide in England [27].

This study aimed to investigate how an ethnically diverse sample of adolescents living in an urban community area spoke about self-harm, and their experiences of disclosure and help-seeking.

## Methods

Thirteen schools from the London Boroughs of Hackney and Newham were invited to participate in this study of self-harm, coping and help-seeking in 2007. These schools were invited as they had previously participated in a longitudinal study on adolescent health [28] which included questions about self-harm. Four schools agreed to participate. Adolescents (aged 15–16 years) in year 11 were given written information about the study for themselves and for their parents. Parent information sheets were translated into Bengali, Punjabi and Urdu to encourage participation in this culturally diverse area. Parents who did not wish for their child to participate could opt him/her out. Adolescents gave written consent during data collection, and could withdraw at any time.

This study used a screening questionnaire administered to 319 participants in secondary schools during school hours to select a sample for individual interviews. Participating mixed ability classes for the questionnaire were selected by each school liaison teacher, based on school timetabling and researcher availability. The questionnaire included questions on demographics and life events [3,28]. Self-reported ethnicity was assessed using an adapted version of the 2001 census question, including ethnic groups prevalent in East London at the time (e.g. Somali and Bangladeshi communities). Categories were also included to allow participants to report identification with having a mixed ethnic identity, such as Asian British, as discussed in research from the RELACHS study [29]. The questions on self-harm were embedded within an adapted version of the A-Cope [30], in which participants were presented with a list of coping strategies they may use when “stressed or upset”, with response options “never thought about it/thought about it, but have never done it/done this once/have done this occasionally/I do this often”. Participants were coded as having self-harmed if they had “taken an overdose” or “harmed yourself in some other way e.g. cut yourself” at least once.

Thirty participants were purposively selected for interview. The aim was to ensure representation across different experiences and frequencies of self-harm, selecting on the basis of repetition of self-harm (never, once, more than once) to include young people who had tried the behaviour without repetition and those who adopted the behaviour and repeatedly hurt themselves. Adolescents who had not self-harmed were interviewed to explore peer attitudes, and to provide insight into potential peer responses to disclosure of self-harm. The sample was selected to include both males and females. Within each school, for every two males or females who had self-harmed, one person of the same gender who had not self-harmed was also invited for interview. Teachers were not given specific detail about the selection criteria. Liaison teachers in each school were given a list of names of those people invited for interviews, and assisted with arranging for the interviews to take place in the school. The selected sample were given information sheets for both participants and their parents, once again giving the parents an option to opt their child out of the study. Participants were invited to the interview, timetabled by the liaison teacher in each school. EK returned to participants’ schools to conduct individual interviews during school hours.

At the start of each interview, each participant was given the original screening questionnaire he/she had completed and was invited to comment on their responses to the questions in the survey as a way of reflecting on stress, coping and self-harm. The interview schedule was flexible to emergent issues and a brief topic guide was used to ensure coverage of key areas, see below. There were no set prompts, allowing the discussion to be influenced by participants’ responses. Notably suicide was not included on the topic guide, and would only have been introduced to the interview if it was raised by the participant. Participants were free to opt out at any stage in the data collection process. A protocol for child protection and risk was in place, with procedures set out to support any participant expressing distress. Participants were given the opportunity to debrief after the interview had ended, and asked to provide feedback on the experience of being interviewed [31]. They were also given information about school-based, local area, community and general sources of help, along with the offer for the research team to liaise with school contacts to initiate discussion of pastoral care, if the participant wished. This approach aimed to maintain participant autonomy, providing them with choices and information. The researcher also had an expert panel to consult, including named mental health professionals and child protection doctors in the local area, if there were concerns about any participants.

Topic guide outlinefor individual interviews

• self-image

• social networks

• stressors

• coping

• self-harm

• social support

• culture

• plans for the future

### Data analysis

Interviews were audio-recorded and transcribed verbatim. The data were stored in Excel, and analysed using a Framework approach [32]. Framework is comprised of five stages, including familiarisation with the data, identification of broad themes, coding and indexing themes within each interview, and charting data into a matrix, with a row of each participant and a column for each theme and sub-theme for analysis. The first five transcripts were reviewed for content by EK and KB. Discussion considered emergent themes, shaping the framework for further enquiry and overall analysis. EK developed the charts directly from the data, with themes reflecting the contents of the topic guide. Ideas emphasised or repeated within the interviews were charted, maintaining language and concepts used by participants. Charts were reviewed and refined through an iterative process.

Thematic categories were defined and refined during the charting process, encompassing meanings attributed by participants to the issues being explored, enabling identification of patterns across and between participants. Earlier transcripts were revisited and coded into themes and subthemes arising in later transcripts to ensure a comprehensive analytical process. Charts were modified until all transcript data was incorporated. This facilitated mapping descriptions of the data, exploring how participants described and presented their accounts of self-harm, noting what they emphasised or downplayed, while identifying convergent and divergent ideas from the adolescents’ accounts of their experiences. Further analysis mined the data with sufficient depth to propose mechanisms of why patterns may have occurred, seeking explanations presented by participants, and comparison across cases to identify patterns within themes. Three transcripts were independently coded by MK to review consistency in coding.

The study sponsor was Queen Mary, University of London. Ethical approval was given by the East London and The City Research Ethics Committee and Research Governance Panels in Hackney and Newham.

## Results

Of the 30 pupils interviewed, ten had never self-harmed, nine had self-harmed on only one occasion, and eleven had self-harmed repeatedly. Table 1 presents characteristics of the interview sample, including ethnic diversity reflecting the local population. The median interview length was 38 minutes (range 17–60 minutes). Interview length was determined by a combination of participant contribution, time taken to discuss the topic guide, and lesson time available in each school. Views from adolescents who self-harmed, including commentary on disclosure and help-seeking will be presented, followed by views from adolescents who had not self-harmed.

**Table 1:** Characteristics of the interview sample, by experience of self-harm

**Characteristics of the interview participants**	**Experience of self-harm**			***Totals***
	**Never (n = 10)**	**Once (n = 9)**	**More than once (n = 11)**	
**Gender**				
Female	7	9	8	*24*
Male	3	0	3	*6*
**Age**				
15 years	8	8	10	*26*
16 years	2	1	1	*4*
**Ethnicity**				
White British & White Other (including UK, Irish, Irish & Welsh, Turkish)	2	1	1	*4*
Asian (including Bangladeshi, Pakistani, Indian and Sri Lankan Tamil)	2	5	5	*12*
Black (including British and African)	3	1	3	*7*
Mixed ethnicity (including White & Black African, African & Asian, White & Black Caribbean, White & Oriental Asian, Pakistani & Asian British)	2	2	2	*6*
Ethnicity not given	1	--	--	*1*

### Talking about self-harm: adolescents who had self-harmed

Participants described a wide range of methods and feelings about self-harm. Forms of self-harm included self-cutting, overdoses, self-battery, punching and self-burning. Some participants described punching walls, hurting themselves in the process, yet did not view that as ‘self-harm’.

*“I just punch stuff, innit? Like punch everything around me and then I get scars.”(Male, 15, Mixed ethnicity, repeated self-harm)*

Although some participants did not describe a specific trigger for their self-harm, most named numerous precipitants, often with one specific trigger for an episode of self-harm. These included challenging relationships, family problems, exams, trouble with schoolwork, and difficulty with feelings such as anger, isolation and frustration.

“if I’ve been really stressed at school and I’m falling behind with schoolwork … but there’s no-one like I could talk to about it, so I would either withdraw from everyone, or cut myself or something like that.” (Female, 15, Black, repeated self-harm)

“It’s like a way of getting your emotions out, it’s focusing on something else, other than what’s making you angry.” (Female, 15, Mixed ethnicity, repeated self-harm)

Participants who had repeatedly self-harmed described a sense of relief, or knowing that hurting themselves would make them feel better. That knowledge was depicted as reinforcement for repetition of self-harm by some participants. Pain was often described as being felt later, rather than while they were inflicting the harm. Others commented that they felt no different afterwards, or emphasised feelings of regret.

*“Seeing the blood pour out, it was just like, yeah, my anger’s going away.”(Female, 15, White, repeated self-harm)*

For some, the emphasis remained on the precipitants of their self-harm. Their problems and feelings at the time overshadowed their injuries.

“I regretted it… It was just like … it was like making myself ugly for no reason, just making my arm look all scarred and everything, no reason, it was just … I just wanted it to be over, basically, what was going on, just to end.” (Female, 15, Black, repeated self-harmed)

Most participants who had self-harmed once described a distance from their experience of self-harm, depicting it as something they had done once in a moment of extreme distress, or something they had experimented with or tried out.

“Well, when I was doing it, I felt OK, it doesn’t matter; other people have tried it, so I might as well, but afterwards it hurts” (Female, 15, Asian, self-harmed once)

“I just find it stupid and just, dumb… I just can’t believe I done that!”

(Female, 15, Black Somali, self-harmed once)

### Suicide and self-harm

Of the participants who had self-harmed, there were six indirect references to suicidal ideation; however none directly reported clear, unequivocal suicide attempts. Ideation was implied through statements such as “*I didn’t feel like I needed to be in the world”*, and the desire for an escape. Comments about having been suicidal were couched within descriptions about what they had done, only after they had done it, acknowledging that their self-harm may have lethal consequences.

*“You don’t see the point of living when you’re upset, it’s like, it’s confusing”(Female, 15, Mixed ethnicity, self-harmed once)*

*“I felt as if I needed a way out, but I couldn’t find one and it was like I was looking for the light at the end of the tunnel, but I couldn’t find it because it was so dark everywhere”.(Female, 15, Asian, self-harmed once)*

Participants were not asked about suicidal ideation by the researcher, so it would only have been included in the interviews if they raised the topic. Suicide was not mentioned by participants who had repeatedly self-harmed. Reasons for this were not clear from this data; however the varying motivations described by participants provide insight into the varied nature of non-suicidal self-injury.

### Self-harm in other people

Participants indicated some difficulty comprehending self-harm in both themselves and in others, irrespective of their own experiences. Some were not aware of anyone else who had hurt themselves. For others, knowing people who self-harmed was integral to their own experiences, normalising the behaviour.

“You don’t start because of friends… when friends do it, it’s like it becomes another option… Sort of like listen to music or reading, it becomes another option. It’s something you just try out.” (Female, 15, Mixed ethnicity, repeated self-harm)

“When I was doing it, I felt OK, it doesn’t matter; other people have tried it, so I might as well, but afterwards it hurts.” (Female, 15, Asian, self-harmed once)

A few participants described being able to cease their self-harm at a time they chose, when they decided to stop, or no longer felt the need to self-harm. However, most reported that cessation of harm was not that straightforward.

### Disclosure and secrecy about self-harm

Most participants described initial reluctance to disclose self-harm verbally or visually. Although participants did speak about their self-harm within the interviews, most had difficulty constructing a coherent narrative or finding words to explain their experiences.

Many did not view self-harm as an issue requiring discussion, preferring to keep their problems and self-harm to themselves. Others justified secrecy with reference to specific people or a fear of negative responses, being labelled a *“weirdo”* or *“getting it thrown back in my face”*.

“It was only important to me that my parents didn’t know, because they would have gone mental.” (Female, 15, Asian, repeated self-harm)

Most participants reported negative experiences when their self-harm had been discovered, reinforcing the desire to maintain secrecy. Shocked reactions from others illustrated attitudes lacking emotional understanding, focusing on the physical injuries. Some participants who self-harmed described having trouble responding to self-harm in others, particularly if they had not hurt themselves for some time.

“I was shocked… because I didn’t know she self-harmed. And it’s like she doesn’t … do it, like how I did it, just to scratch; she actually like does it a lot and I was like … OK. I didn’t really know what to say to her.” (Female, 15, Asian, self-harmed once)

Two participants wanted others to view their self-harm; cutting themselves with the purpose of being seen. One described cutting herself in front of her brother to provoke a reaction, looking for signs of care. The other described using his self-harm as a means of communicating and seeking help.

“The thoughts I used to have… after cutting myself and before cutting myself, were, like, just show somebody… I wasn’t a good talker, like, back then, so … that’s why I knew that they would kind of help me in some way.” (Male, 15, Asian, repeated self-harm)

Some of the participants who had ceased self-harming viewed disclosure more positively with hindsight. Those adolescents could see how disclosure of their harm led to changes in the problems they were facing, feeling more understood or reassured after others found out.

“Actually it did make me feel better, like, I knew that people did love me and it made me feel better that someone else knew. I didn’t want them to know, but it made me feel better in a way.” (Female, 15, Black, self-harmed once)

### Help-seeking

As most participants had never sought help, discussion of help-seeking related to their ideas about it, rather than experiences. These participants did not have a clear notion of where they might go for help, nor what to expect. Some participants suggested that people who were similar to them in age or background may be easier to talk to, whereas others expressed a desire only to talk to people who seemed different from them; not being of the same background or culture, not living in the same area or being involved with their school. Doctors and nurses were only mentioned by those who had received medical assistance (and those who had not self-harmed). Some participants commented that as teenagers, they might have difficulty seeking medical help without involving family members. Teachers and school staff were seen as accessible sources of help, yet not trusted as they were often from the local community and were predicted to share the disclosure with others. Barriers to seeking formal help included scepticism about the potential responses participants may receive, fear of the potential consequences of seeking help at school or it becoming more known in their local or cultural community. Others simply did not view their harm as problematic.

*“I don’t think they should contact any sort of outside help, unless the student wants it. Because if the student’s getting it, but doesn’t want it, it’s not going to help.”(Female, 15, White & Asian, repeated self-harm)*

If they were going to seek help, participants hoped for confidentiality and respect. As many accounts of self-harm revealed situations and feelings functioning as triggers, participants wished for help to address those precipitating factors. Examples included reducing conflict or responsibilities within their families, easing schoolwork pressures or having places to go if feeling distressed.

“…*understanding, like where I’m coming from sort of thing”(Female, 15, Black, repeated self-harm)*

Having a trustworthy person to talk to was suggested as a way to reduce the desire to self-harm, consistent with descriptions of feeling isolated as a trigger.

“if you had someone there … it wouldn’t come to your mind to do those things, but it’s at a time when you … when kids have no-one at all that you would do the craziest things, and not care at all how it hurts you” (Female, 15, Black African & Asian, self-harmed once)

### Response to self-harm without help being sought

Due to the public nature of some self-harm, help was sometimes offered without it being sought. For example, if physical evidence of self-harm had been noticed, responses from others may have included referral for further help. An extract from an interview with a participant is presented below, describing her experiences. Being “found out” was not unusual. She described the knowledge of her self-harm being passed on, removing her control over who knew about it. There was an awareness that friends or teachers would hold different views on self-harm, and that responses may be influenced by the roles different people played in the school and wider community.

Example of a response to disclosure of self-harm and being given help

Extract of interview between a 15 year old female, Black British participant (P) and the interviewer*(I).*

**P:** I told my friend. My mum didn’t know at first. Because the school … the school found out because when I done P.E. I had short sleeves on and then my friend saw it. And then someone went and told my head of year, that I didn’t know about, and then they found out and then they told my mum*…*

….[I: How did you think it was handled by the school?]

**P:** It was OK. I didn’t mind the way it was done, but, I don’t know. Yeah. It was all right…

[I: … what’s helpful that someone at school could do?]

**P:** I don’t think the school can do very much, because they’re just doing their job, basically. That is within school hours; outside school, they’re not really going to do much for you. The only thing they’ll do is just tell family or they’ll get social services involved, but I don’t think they can do much for you.

[I: Uh huh. I’m also looking at how they do it… so like you said, when you were in P.E. when your friend told a teacher, how did you feel about that?]

**P:** At first I was angry, because… I didn’t know she told, so I was like, “Why didn’t you tell me?” But then she was just upset at the fact, what I was doing, so she didn’t really care how I felt. It was she just wanted me to get help, sort things out.

[I: So when she told a teacher, what happened next? Did they come and find you, or…?]

**P:** They wanted to see my hand. And then they asked me why. And then my form tutor knew, and my head of year knew and another teacher that deals with … I don’t know what she is really; like I think she deals with people’s behaviour problems and things like that.

[I: Uh huh. How did you find it when all these people knew?]

**P:** I was like, “How did you know?!” So much people, like, a lot! I was just amazed how they knew, because I didn’t tell any of them. But then the word just got passed around. And then they were trying to … it’s like they were trying … they didn’t want to get me angry. It was like they were treating me like I had a disability, I didn’t really like it; they wasn’t treating me like they would normally treat me if they didn’t know that I was harming myself.

The need for appropriate help was illustrated in these accounts, with emphasis on the importance of accessibility and privacy, especially in close-knit communities or if family was involved in the help-seeking process.

“But it’s hard, like… my mum watching or my brother watching me, or someone like that. So it’s kind of hard to say, call up and speak to someone in front of somebody else, when it’s supposed to be confidential… So, I think if they are on-line, probably just emailing or talking to someone online … that’s better.” (Female, 16, Asian, self-harmed once)

### Talking about self-harm: adolescents who had not self-harmed

These participants expressed a range of views, including disparaging comments, emphasising*“attention-seeking”* and distinguishing between *“proper personal issues”* and self-harm *“just being stupid”.* Adolescents who had never hurt themselves also associated self-harm with suicide.

*“I don’t like people who purposefully like try and attention seeking and like who go to hospital and waste doctors’ time … I think if you’re going to do it, yeah, do it properly, yeah? If you really want to hurt yourself, die or whatever, then just do it, yeah?”(Female, 15, Mixed ethnicity)*

Participants who had never self-harmed conceded that without having intentionally hurt themselves, they may not be able to comprehend reasons to do so. It was suggested that helping someone who had self-harmed could be challenging, without understanding or having shared their distress.

“I’m not in that person’s shoes. So I can’t speak on behalf of that person. I don’t know what that person’s going through.” (Female, 15, White UK)

Two participants who had never self-harmed spoke of it as acceptable as long as it was hidden. This emphasis on self-harm as a personal, individual act corresponds with similar comments by those who had self-harmed. Such views may reinforce the perceived need for secrecy about self-harm.

“I just think she’s attention seeking, because… if someone really had to self-harm and they felt that low, they wouldn’t be expressing it to everyone else, because they’d feel so bad within themselves. They wouldn’t be like putting on a show like, “Oh, I have to do this, because I feel so bad.” You’d do it and you’d keep it private.” (Female, 15, Mixed ethnicity)

## Discussion

This study presents unique descriptions of how adolescents living in urban England view self-harm. Adolescents depicted self-harm as a complex behaviour involving a range of methods, functions and taboos. Those who self-harmed described it as a private, inwardly focused expression of distress, often with a reluctance to disclose and seek help. This was reinforced by the comments about self-harm as attention seeking from participants who had not self-harmed. Such views may contribute to fears about responses from others, particularly where social support may be variable or lacking. Self-harm being discovered by others was often viewed as a negative experience. Although most participants were unclear about what would constitute “help”, some reflected on the benefits of help they had received following disclosure of self-harm. The ethnic diversity within this sample illustrated that self-harm was concern across a range of ethnic groups. Mixed reflections and experiences described in this study elucidate feelings and intentions which may accompany non-suicidal-self-injury or other self-injurious behaviours.

There were inherent challenges in exploring retrospective accounts of self-harm. The difficulty participants had constructing a coherent narrative about self-harm experiences and intentions may relate to a general difficulty expressing their feelings, poor memory of the events or feeling uncomfortable talking about it. Alternatively the sense of dissociation during self-harm described by some participants may explain patchy recollections and a perceived lack of pain. It is feasible that given the intense emotional states reported when describing self-harm, intentions may not always be apparent to the individual after the event, and may go unreported in retrospect.

This qualitative research presents insights on a sensitive topic from a hard to reach community sample, which complements the findings from survey-based research [17] particularly regarding the social context for self-harm and help-seeking. The inclusion of adolescents both with and without personal experience of self-harm provides information on the attitudes adolescents who self-harm may encounter in their peers. Previous research highlights the association between self-harm and the absence of confidantes [33,34], supported in this study through discussion of the isolated nature of some self-harm. That is, perceived isolation may increase the difficulty seeking help.

Analysis suggests that help-seeking may be a more dynamic process than “staged” models of help-seeking indicate [35]. Reasons to seek help (or not) may change over time, alongside changing perceptions of the problem, the context and throughout the process of seeking help. Difficulty in seeking help may arise due to the distressing nature of self-harm, potentially including suicidal ideation, or if the adolescent believed that their disclosure would lead to negative consequences. This is of great concern, akin to the help-negation effect, where higher suicidal ideation has been associated with reduced intentions of seeking-help in young people [15].

The raw nature of the accounts presented in this study may resemble communication in the presentation of self-harm to clinicians or other sources of help, and illustrates the variation in the circumstances and interpretations of self-harm by adolescents. Comments from adolescents who had not self-harmed provide evidence that help-seeking may also be challenging for those being asked for help. This complements research on help provision for young people who self-harm being difficult for teachers [36] and healthcare professionals [37,38]. Findings illustrate the complexity required for providing support that is both sensitive and acceptable to adolescents who hurt themselves. Engaging adolescents with services may prove difficult, particularly if self-harm was not viewed as problematic or worth discussing. Personal accounts emphasised the need to address issues leading to self-harm, and not solely respond to the physical injuries. Practitioners may need to consider social, family and cultural influences on an adolescent’s life when endeavouring to provide help for self-harm.

This study highlights potential areas of training and development to promote accessible and acceptable care for young people. For example, one-stop-shop facilities provide multiple services in one place to increase the acceptability of service use, if the nature of help being sought could remain concealed. Online service access for young people may overcome barriers relating to privacy and being physically unable to visit a service. Culturally appropriate services may reduce barriers to help-seeking in ethnically diverse communities, particularly as it is known that culture influences causal explanations of health and health behaviours [39]. Barriers may also be reduced if service providers were trained to feel more confident in being able to respond appropriately, or even to open discussions, taking initiative to help young people who may not be comfortable taking that step alone.

Findings about lack of understanding about self-harm, and difficulty responding to self-harm in others raises the need to promote awareness about emotional health, coping strategies and sensitivity in responding to distress in oneself and others at a community level [3,40,41].

Public health interventions in schools to train teachers and welfare staff may facilitate discussion of such issues more routinely, increasing awareness of available support, facilitating help-seeking by making talking about distress less taboo [42], increasing confidence and knowledge in staff working with young people who self-harm [43] and aiming to ensure help-seeking would not increase stigma for young people [41]. Treatment of anxiety and depression in adolescents who self-harm may alleviate future distress, potentially reducing the risk of later self-harm and suicide [4]. There is also scope to promote easily accessible support in schools and the community, given the constraints adolescents may perceive about help-seeking.

### Strengths and limitations

This qualitative exploration with a young, hard-to-reach sample provides insight into the social and cultural context of adolescent self-harm. Interviews with 30 young people is a strength of the study, facilitating in-depth probing of a wide range of views and experiences of self-harm, and detailing the variability in attitudes accompanying this complex behaviour. However, there are limitations in the insights obtainable from a study using a single interview with adolescents. Given the sensitivity of the issue, follow-up interviews may have enabled adolescents who were more reticent in speaking about self-harm or who had difficulty constructing a coherent story from their recollections to add further perspectives*.* Future research could explore issues arising in this study in more depth, such as social influences on adolescent self-harm [6], comparison of experiences in urban, rural and international settings, experiences throughout the process of accessing help, and the potential for psychosocial factors to play a protective role [44]. Further investigation into adolescent self-harm could explore the nature of cultural influences [45] and experiences reported by adolescents from different ethnic groups. There is scope for exploration and interventions for both giving and receiving help; how to make help more acceptable and accessible to adolescents who self-harm and to identify preventive interventions which might help adolescents find more adaptive ways to cope with distress. Although epidemiological research shows a natural decline in self-harm as adolescents reach early adulthood [4], the subjective distress articulated by young people who self-harm demonstrates the need to provide support accessible to adolescents at a community level.

## Conclusions

This qualitative exploration of youth perspectives on disclosure and help-seeking in self-harm presents a range of views highlighting the complexity and diversity of this adolescent experience. Disclosing self-harm can be difficult, in part due to the potential impact of responses from others. The findings of this study can be applied to inform public health interventions aiming to increase awareness and sensitivity about responding to self-harm, the emotional wellbeing of young people and in those who support and work with them.

## Competing interests

The authors declare that they have no competing interests.

## Authors’ contributions

This work was completed as a part of EK’s PhD, supervised by KB and SS. Ethical approval was granted to EK, KB and SS. Data was collected and analysed by EK. MK and KB advised on qualitative analysis. All authors contributed to the writing of the manuscript, read and approved the final manuscript.

## Authors’ information

EK is the inaugural Marie Bashir Fellow in Adolescent Medicine in the Academic Department of Adolescent Medicine, University of Sydney. MK is employed as a Senior Lecturer by Queen Mary University of London.

## Pre-publication history

The pre-publication history for this paper can be accessed here:

http://www.biomedcentral.com/1471-2458/13/572/prepub
